# Biomarkers to Monitor Adherence to Gluten-Free Diet by Celiac Disease Patients: Gluten Immunogenic Peptides and Urinary miRNAs

**DOI:** 10.3390/foods11101380

**Published:** 2022-05-10

**Authors:** Alessandro Paolini, Meysam Sarshar, Cristina Felli, Stefania Paola Bruno, Mohammad Rostami-Nejad, Francesca Ferretti, Andrea Masotti, Antonella Baldassarre

**Affiliations:** 1 Research Laboratories, Bambino Gesù Children’s Hospital-IRCCS, 00146 Rome, Italy; alessandro.paolini@opbg.net (A.P.); meysam.sarsharjeryandeh@opbg.net (M.S.); cristina.felli@opbg.net (C.F.); stefaniapaola.bruno@opbg.net (S.P.B.); antonella.baldassarre@opbg.net (A.B.); 2 Department of Science, University Roma Tre, 00146 Rome, Italy; 3 Gastroenterology and Liver Diseases Research Center, Research Institute for Gastroenterology and Liver Diseases, Shahid Beheshti University of Medical Sciences, Tehran 1985717411, Iran; m.rostamii@gmail.com; 4 Hepato-Gastroenterology and Nutrition Department, Bambino Gesù Children’s Hospital-IRCCS, 00165 Rome, Italy; francesca.ferretti@opbg.net

**Keywords:** circulating miRNAs, fecal miRNAs, urinary miRNAs, gluten immunogenic peptides, GIPs, Celiac disease, gluten-free diet

## Abstract

Celiac disease (CD) is a multifactorial autoimmune enteropathy with a prevalence greater than 1% in the pediatric population. The only therapy for CD patients is a strict gluten-free diet (GFD). Gluten-free food contamination by other cereals during packaging and cooking or accidental ingestion of gluten may cause several intestinal and extraintestinal symptoms in CD patients. Therefore, the monitoring of gluten contamination in food and assessing the level of ingested gluten by analytical biomarkers has been of great interest in recent years. To this aim, small gluten immunogenic peptides (GIPs) obtained by the hydrolysis of gluten and present in urine and feces have been studied as biomarkers of gluten intake and to monitor adherence to GFD by CD patients. More recently, the use of circulating, fecal and urinary miRNAs has emerged as a novel diagnostic tool that can be potentially applied to assess adherence to GFD. Moreover, the presence of GIPs and miRNAs in both feces and urine suggests a similar excretion modality and the possibility of using urinary miRNAs, similarly to GIPs, as potential biomarkers of GFD in CD patients.

## 1. Introduction

Celiac disease (CD) is a multifactorial immune-mediated enteropathy distributed worldwide with a prevalence of approximately 1% [1], although significantly higher in children [2], that is triggered by environmental and genetic factors [3]. CD exhibits a wide spectrum of clinical, serological and histological manifestations. The environmental factor responsible for its onset and maintenance is gluten, a complex mixture of protein contained in grains, such as wheat, rye, oat and barley. Gluten includes two major protein types: gliadins and glutenins (hordeins and secalins in barley and rye, respectively) [4]. Gliadins and glutenins have a high proline and glutamine content and are resistant to gastrointestinal enzymatic proteolysis. Therefore, large and potentially immunogenic peptides are prevalent on the intestinal mucosal surface [5].

Depending on their electrophoretic mobility, gliadins are categorized α, β, γ or ω-gliadins, whereas glutenins are categorized as either high- or low-molecular-weight subunits [6]. Overall gluten proteins are known as prolamins, and although they are present in all cereals, only those of wheat, barley and rye are harmful for individuals susceptible to CD. The prolamin fractions contained in barley and rye are referred to as hordeins and secalins, respectively. Nevertheless, the prolamin fractions of oats (i.e., avenins) share some common sequences with gliadin, and evidence showed that a small subset of CD patients can become intolerant to oats (most likely owing to wheat, barley or rye contamination) [7]. Cereals that are not harmful to CD patients can be employed in a GFD, and this diet generally includes rice (oryzeins), maize (zeins) [8] and buckwheat (albumins and globulins) [9].

The genetic factor is the presence of specific HLA alleles in CD patients. In particular, it has been observed that 90–95% of CD patients express HLA-DQ2, and the remaining 5–10% express HLA-DQ8 [10]. In these genetically-susceptible individuals, the ingestion of dietary gluten induces inflammatory processes and small-intestine mucosal injury characterized by villous atrophy, crypt hyperplasia and infiltration of many lymphocytes and plasma cells in the *lamina propria* [11]. A 33-mer peptide was identified, and its characteristics have led to it being considered the primary initiator of the inflammatory response to gluten in CD patients [12]. All of these events affect the gastrointestinal tract, and the most common symptoms displayed by CD patients are malabsorption, diarrhea, weight loss and nutritional deficiency. However, CD patients can also experience extraintestinal symptoms, such as anemia, osteoporosis or arthritis.

The presence of highly specific autoantibodies for CD, such as anti-tissue transglutaminase IgA (TGA-IgA), anti-endomysium IgA (EMA-IgA) and anti-deamidated forms of gliadin peptide IgG (DGP-IgG), circulating in the blood of CD patients as a consequence of the immunological response to gluten can be employed to evaluate adherence to GFD and exposure to gluten [13,14], although they have less than 50% of sensitivity to detect villus atrophy [15]. The gold standard for the diagnosis of CD is a combination of serology and duodenal biopsy. In 2012, the European Society for Pediatric Gastroenterology, Hepatology and Nutrition (ESPGHAN) guidelines suggested to avoid biopsies in children/adolescents with HLA-DQ2/DQ8 haplotype, those who are EMA-IgA-positive and those with TGA-IgA levels greater than ten times the normal limit [16]. The new guidelines released in 2020 confirmed the safety of the ‘no-biopsy’ approach for the diagnosis of CD in children with high levels of TGA-IgA and positive EMA-IgA in a second serum evaluation [17].

Until now, the only available treatment for CD has been lifelong adherence to a gluten-free diet (GFD) [18]. For many years, clinicians and researchers discussed the sensitivity, specificity and accuracy of the many available serological tests, such as TGA-IgA, EMA-IgA and intestinal fatty acid binding protein (I-FABP) or citrulline [19], employed to monitor the response to GFD without reaching a conclusive consensus on a particular test. It is also true that the adherence to GFD by performing repeated intestinal biopsies is not feasible or practical [20,21,22].

Recently, gluten immunogenic peptides (GIPs) and circulating microRNAs (miRNAs) have emerged as valuable candidate biomarkers to monitor not only the presence of gluten in feces and urine (i.e., the GIPs) but also to diagnose several diseases, such as CD (i.e., circulating miRNAs) [23]. 

Previous works reported the dysregulated presence of miRNAs in blood [24] or plasma [25] of CD patients compared to controls or GFD patients. Some authors identified miRNAs as potential early predictors of CD before its development [26], whereas others failed to report consistent and statistically significant data about circulating miRNAs in CD patients [27].

In this review, we will provide a comprehensive overview of the presence and use of GIPs in feces and urine of CD patients and their use as biomarkers of gluten ingestion. We will also provide some comments and a brief discussion on the potential of urinary miRNAs as novel biomarkers of adherence to GFD in CD patients, as already demonstrated for other diseases.

## 2. Monitoring Adherence to GFD

Adherence to a strict GFD is the only treatment available for CD patients to date. Therefore, following diagnosis of CD, it is important to monitor adherence to GFD to prevent intestinal symptoms and minor damage [28]. However, gluten is ubiquitous, food cross contaminations are possible, food labeling is sometimes inadequate and all of these issues make it difficult for CD patients to strictly adhere to GFD [29]. It has been reported that most CD patients consume measurable amounts of gluten, although they are on a GFD, and that there is a discordance between gluten detection and villous atrophy, as consumption of as much as 10 mg of gluten daily seems to be tolerated by CD patients [30], whereas higher amounts (i.e., 200 mg/day) trigger symptoms and sustain intestinal damage [31].

To date, the most commonly used method to assess adherence to GFD consists in dietary questionnaires, serological tests or the evaluation of clinical symptoms, although none of these methods can directly and accurately estimate adherence. In fact, patient self-reporting appeared to be subjective and inaccurate in terms of the level of adherence to GFD or the lack of reporting the intentional or unintentional consumption of gluten [28,32]. The evaluation of serum levels of CD-specific antibodies plays a crucial role in monitoring adherence to a GFD diet, and numerous studies report that high levels of TGA-IgA or DGP-IgG may suggest non-adherence to the diet [33,34]. However, this method has limitations, as the normalization of antibody titers is lengthy, and it cannot identify sporadic episodes of occasional gluten exposure [28].

Small intestine biopsy is the most accurate method for diagnosing CD and its severity [35] and could be used to monitor adherence to GFD. However, this procedure is invasive, expensive and not particularly practical for this purpose [36]. Finally, the persistence of gastrointestinal symptoms after prolonged GFD may not be the evidence of non-adherence to the diet, as patients under strict GFD showed more symptoms than healthy subjects [37]. Moreover, bacterial overgrowth in the small intestine, irritable bowel disease or refractory CD can develop in subjects on a GFD, demonstrating the inefficacy of symptom monitoring [28].

For all of these reasons, the discovery of novel molecules able to function as biomarkers of GFD is particularly relevant and urgently needed. We think that GIPs and miRNAs could fulfill this role and that they can be used as complimentary biomarkers to monitor GFD in CD patients.

## 3. Production of GIPs by the Hydrolysis of Gluten

Prolamins (i.e., glutelin, glutenin and gliadin) are a group of proteins containing a mixture of many different subunits that range from 18 to >100 kDa. They are the main components of gluten, with a great quantity of proline- (P) and glutamine (Q)-rich proteins (although poor in lysine), mainly found in the seeds of cereal grains, such as wheat, barley, rye, corn, sorghum and oats [4]. The capacity of P to fold in secondary structures favors the storage of amino acids required during germination of the seeds, but at the same time represents an obstacle for efficient hydrolysis. Under normal conditions, upon ingestion, glutamine- and proline-rich proteins are hydrolyzed by proteases of the gastrointestinal tract [5], but repetitions of P residues in the protein sequence prevent gastrointestinal enzymes from hydrolyzing this polypeptide at the position immediately next to P. The result of this ‘hindered’ hydrolysis is the formation of a mixture of large peptides. The size of these digested peptides, along with the position of the Q residues in the primary structure of the peptides, plays a pivotal role in their activity in CD [8,38]. The principal component able to activate CD in patients is α-gliadin, which is a peptide with 277 amino acid residues. Once the α-gliadin reaches the *lamina propria* (i.e., the mucosa) by transcellular or paracellular transport, it is processed by tissue transglutaminase (TGA). The result of the digestion of gluten is a relatively large fragment, referred to as the 33-mer (i.e., LQLQPFPQPQLPYPQPQLPYPQPQLPYPQPQPF), which maps the 57–89 N-terminal region, which is resistant to further gastrointestinal breakdown. The sequences derived from the α-gliadin fraction of gluten are referred to as GIPs. In CD patients, generally bearing a distinctive HLA-DQ2 or HLA-DQ8 genotype, these peptides are toxic and immunogenic [39]. In fact, GIPs are presented by antigen-presenting cells (APCs) to TCD4^+^ lymphocytes, which trigger a cascade of several immune responses with the production of interferon-γ and IL-15 that lead to villi atrophy, crypt hyperplasia and increased tissue infiltration of lymphocytes [4].

Recently, the determination of GIPs in feces and urine has emerged as a tool to diagnose the accidental ingestion of gluten and to predict adherence to a strict GFD or help clinicians to assess the adherence [40].

In the following paragraphs, we will discuss in detail the use of GIPs as biomarkers of gluten ingestion in CD patients.

## 4. Detection of GIPs in Feces

Due to resistance of GIPs to gastrointestinal digestion and the potential immunogenic reactions of TCD4^+^ cells in CD patients, the clinical usefulness of fecal GIPs quantification has been recently evaluated as a marker of dietary transgression/adherence to GFD [12,41,42,43,44,45] or to detect duodenal mucosal damage in CD patients [46].

Comino and colleagues were the first to detect gluten-derived peptides in feces of patients with CD or other intestinal pathologies [12]. They found that the recovery of immunotoxic fractions in feces could indicate that gluten has passed through the digestive tract because a significant portion of the ingested gluten peptides was excreted. Therefore, gluten has been consumed and could be measurable in feces. Additionally, the concentration of 33-mer peptide was determined using in vitro simulated gastrointestinal digestion. Their results indicate that more than 30% of the gliadin-reactive peptides remained undigested, even after hydrolysis during digestion. The variations in the absorption of intact gliadin-reactive peptides between individuals could explain the diversity of the microbial population in the gastrointestinal tract, which might impact the peptide concentration in feces [12,47]. In a further comprehensive study, the authors evaluated the efficacy of fecal GIPs to support the diagnosis of the disease and monitor adherence to GFD in a cohort of CD children. Their analysis of fecal GIPs resulted in two main findings: the direct confirmation of gluten intake days before CD diagnosis and a substantial decrease in gluten consumption after diagnosis of CD and the beginning of GFD [48].

Prior prospective, nonrandomized, and multicenter studies performed by the same group revealed the effectiveness of fecal GIPs in 188 CD patients who were on a GFD for at least 1 year versus 84 healthy controls. Fecal GIPs quantification by measurement of serological anti-tissue transglutaminase IgA (TGA-IgA) and anti-deamidated gliadin peptide IgG (DGP-IgG) antibodies was performed simultaneously. The authors found a significant association between age and GIPs in feces with an increasing dietary transgression with advancing age and with gender in certain age groups: 39.2% in subjects ≥13 years old and 60% in men ≥13 years old [32].

With respect to the traditional dietary questionnaire or serological methods, their analyses showed a low GFD adherence rate among patients on an established GFD using GIPs analysis. These findings suggest that the fecal GIPs analysis can be an accurate method that enables a direct and quantitative assessment of gluten exposure soon after ingestion. However, a combination of GIPs tests with serological tests did not show a good correlation and remained a complementary technique.

Other authors assessed dietary adherence with the Biagi questionnaire and serum TGA-IgA of 55 consecutive CD patients (27 adults and 28 children) who had been on a GFD for at least 2 years. GIPs were determined using immunoenzymatic assay, and their results showed that fecal GIPs were detectable in 14.5% of CD patients who had been on a GFD for at least 2 years. Moreover, their combination of Biagi score and fecal GIPs revealed promising assays for monitoring dietary adherence [43]. The authors concluded that the most important and useful aspects of GIPs detection are involuntary gluten intake monitoring, explaining the persistence of symptoms; positivity of serum biomarkers; and reduction in unnecessary and invasive biopsies. All of these aspects make the detection of fecal GIPs a promising tool in the management of CD patients [43].

In a cross-sectional and prospective study conducted by Roca and colleagues, fecal samples from CD children were analyzed by rapid immunochromatographic (IC) and ELISA tests, both based on the G12, the anti-gliadin 33-mer monoclonal antibody. A total of 67 CD children were grouped as either children on a GFD (43 cases) or neo-diagnosed children (18 cases). Their results showed that according to the food records, 39 out of 43 patients were compliant with the GFD, and gluten consumption was recorded in 4 children. GIPs were detected in 15/43 individuals by the ELISA method and in 7 individuals by IC strips. However, GIPs levels decreased significantly over time in a non-linear way after starting GFD and were below the detection limit on the third day in most individuals [42]. This group has previously obtained high specificity (100%) for both IC strips and ELISA in detecting GIPs in feces of healthy infants [44].

It has been recently reported that GIPs quantification in feces indicates dietary transgressions in patients on long-term GFD according to dietary reports. Costa and colleagues followed-up CD adult patients who were on a GFD for at least two years. They found that in 67 out of 74 (90.5%) fecal samples, both ELISA and point-of-care tests (PoCTs) were concordant (either positive or negative) [49]. Integration of GIPs quantification in feces with conventional strategies could better determine adherence to GFD both in children and in the adult population. Altogether, the more gluten consumed, the higher the detectable concentration of GIPs in feces of CD patients on a GFD; however, the presence of several gastrointestinal factors that could influence GIPs recovery cannot be ruled out and needs to be further investigated.

## 5. Detection of GIPs in Urines

Similar to fecal GIPs, the presence of GIPs in urine has been investigated in recent years to evaluate the potential toxic effects of gluten peptides contained in food accidentally ingested by CD patients [8]. The reliability of the quantification of urinary GIPs was analyzed in a randomized, double-blind study in which a cohort of healthy children were exposed to different amounts of gluten. The detection of urinary GIPs occurred immediately and after 24 h, in comparison to a second cohort that underwent a strict GFD. The performance of an IC assay was dose–response-dependent. In fact, the recovery of GIPs in urine increased in parallel with the amount of ingested gluten [50]. Similarly, Coto and colleagues assessed the lateral flow immunoassay on urine excreted by 20 healthy participants challenged with 50 mg and 2 g of gluten ingested for 8 and 12 days, respectively. The amount of detected GIPs exhibited a time-course correlation with the maximum excretion centered about 6–9 h after ingestion [41]. A recent study by Moreno and colleagues reclassified patients diagnosed with refractory celiac disease (RCD) only by evaluating GIPs in urine [51]. In particular, the authors were able to distinguish between individuals ‘exposed to gluten’ and RCD patients who had instead intestinal inflammation but a clear absence of gluten exposure.

Ruiz-Carnicer and colleagues investigated the clinical utility of GIPs as biomarkers to monitor adherence to GFD and the relationships between the detectable levels of GIPs in urine and the degree of damage to the intestinal mucosa [52]. About 24% of CD patients on GFD still had mucosal damage, and 94% of them had detectable urinary GIPs. In contrast, 97% of patients without duodenal damage had no detectable GIPs. The authors demonstrated the high sensitivity (94%) and negative predictive value (97%) of GIPs measurements in relation to duodenal biopsy results. In addition, the authors demonstrated that in the group of neo-diagnosed CD patients, 82% had measurable amounts of GIPs in their urine.

In another similar study, urine samples were collected from 76 healthy subjects and 58 CD patients on different dietary regimens (and therefore gluten amounts). Data indicated that 89% of CD patients without villous atrophy had no detectable GIPs in the urine, whereas those patients with a damaged intestinal mucosa exhibited a detectable amount of GIPs in their urine, thus demonstrating a good correlation between the presence of GIPs in urine and increased damage to the intestinal epithelium [53].

However, different conclusions were reported in a recent paper, where the authors compared the results achieved through the administration of a food questionnaire and studied the quantification of GIPs excreted in feces and urine. They demonstrated that in their cohort of 70 subjects, fecal GIPs was a useful biomarker to assess gluten intake, but urinary GIPs were not detected at all [45].

## 6. Urinary miRNAs as Biomarkers of Kidney Diseases

Urine represents one of the best biofluids for clinical diagnostic practice, owing to the easy collection procedure, non-invasiveness, possibility of collecting large amounts and the absence of significant proteolytic degradation compared to other biofluids. Moreover, urine has a simple composition compared to serum or plasma, which allows for easy isolation and identification of biomarkers [54]. Despite this, urine contains not only miRNAs, long noncoding RNAs (lncRNAs) and messenger RNAs (mRNAs) but is also an excellent source of many other molecules (i.e., peptides, exosomes, cell-free DNA, methylated DNA and circulating tumor cells) that can be used as potential biomarkers [54].

**Figure 1:**
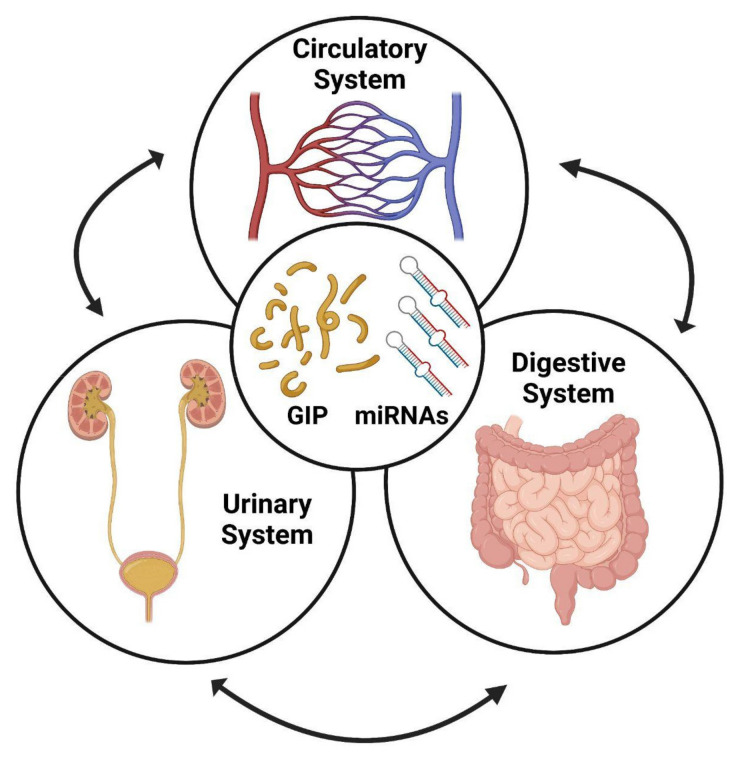
Schematic representation of the links between circulating, fecal and urinary miRNAs and the possibility of detecting GIPs and miRNAs in serum, feces and urine as biomarkers of adherence to GFD in celiac disease (CD) patients. This image was created with BioRender.com.

It has also been reported that patients with gastrointestinal disorders are often associated with an increased risk of developing several kidney diseases [55]. Moreover, we would like to focus on the relationships (if any) between miRNAs released in circulation (i.e., circulating miRNAs) and the miRNAs released by the kidney in urine (Figure 1). With an aim of discussing mainly human clinical studies, we extended the already published review paper [56] with updated findings.

In recent years, there has been a growing interest in urinary exosomal miRNAs in the pathogenesis of diabetic nephropathy (DN). DN is the most frequent form of chronic kidney disease and is a major complication of both type 1 and type 2 diabetes (T1D and T2D). Concomitant T1D or T2D predisposes CD patients to severe comorbidities, as well as to poor dietary adherence [57]. DN is characterized by a progressive deterioration of renal function that can lead to end-stage renal disease (ESRD) [58]. Albuminuria is currently widely used as a biomarker for DN. However, recent studies suggest that microalbuminuria is not a precise predictor of DN risk, and its sensitivity and specificity for early disease detection are limited [59]. Therefore, the identification of biomarkers reflecting early effects during disease development and progression has important clinical value because it would allow for better management during the clinical course of this pathological process.

Urinary miRNAs can provide a possible alternative to currently available biomarkers. In fact, the significance of miRNA in DN has been explored in several studies. The first evidence that the miRNA profile is altered in urinary exosomes from type 2 DN patients (14 miRNAs upregulated and 2 miRNAs downregulated) was provided a few years ago [60]. Principal component analysis revealed that differential urinary exosomal miRNA expression is different in patients with microalbuminuria compared to normo-albuminuric patients. The increased expression of miRNA-320c, which is indirectly involved in TGF-β signaling via targeting of TSP-1, may represent a novel candidate biomarker for early progression of disease.

Eissa and colleagues identified three novel urinary miRNAs (miR-133b, miR-342 and miR-30a) that can be considered biomarkers of DN and also be viewed as potential non-invasive biomarkers for type 2 diabetes. These miRNAs were altered not only in micro- and macroalbuminuria groups but also in some normo-albuminuria cases prior to albuminuria [61].

Gonzalez and colleagues presented the largest human study to date to identify miRNA biomarkers of DN and lupus nephritis (LN) associated with both histopathological lesions on kidney biopsy and functional markers of kidney damage. Five miRNAs in DN and four miRNAs in LN were validated in independent multi-institutional cohorts when compared with healthy and disease controls. Three DN miRNAs (miR-2861, miR-1915-3p and miR-4532) and two LN miRNAs (miR-3201 and miR-1273e) showed a strong ability to discriminate between patients with renal disease vs. either healthy or disease controls and were associated with the most characteristic histopathological lesions. In addition, they confirmed the expression of four out of five of these top miRNA candidates in healthy kidney tissue. These results identified new urinary miRNAs and suggested their use as sensitive and specific noninvasive biomarkers carrying a histopathologic signature of DN or LN and avoiding the use of invasive tests [62]. Similarly, to diagnose renal fibrosis in LN, urinary miR-21, miR-150 and miR-29c were found to distinguish low chronicity index (CI) from moderate–high CI in LN patients with a high sensitivity and specificity (94.4% and 99.8%) and to predict an increased risk of progression to end-stage renal disease (ESRD) [63].

Argyropoulos and colleagues demonstrated that urinary miRNA profiles in T1D patients differ across the stages of diabetic nephropathy. They found changes in specific miRNAs (27 differentially expressed miRNAs) involved in specific pathways known to be altered in various forms of renal disease. They suggested that a number of miRNAs in urine may serve not only as molecular signatures of different clinical phenotypes in DN but also as early indicators of alterations in specific biological processes in the kidney [64]. In another study, the same authors reported differences in miRNA profiles in T1D patients without nephropathy and patients who subsequently developed microalbuminuria. The predicted targets of these miRNAs belong to biological pathways known to be involved in the pathogenesis and progression of diabetic renal disease. Furthermore, these studies demonstrated, for the first time in diabetes, the differences existing in the miRNA profiles between male and female patients [65]. Aimed at identifying specific candidate biomarkers of DN in diabetic patients, a recent study explored the pattern of miRNA expression in kidneys and urine collected from diabetic patients with different types of histologically defined renal damage. Results identified two novel miRNAs that are able to discriminate different histological lesions in diabetic and chronic kidney disease patients. The downregulation of miR-27b-3p and miR-1228-3p, initially detected at the tissue level and in urine, suggests that miR-27b-3p and miR-1228-3p represent two possible non-invasive urinary candidate biomarkers for DN diagnosis and prognosis [66]. El-Samahy and colleagues evaluated the carotid intimal thickness (CIMT) in pediatric patients with T1DM and its association with DN. Their results showed that urinary miR-377 and miR-216a are potential biomarkers of vascular damage [67,68]. Finally, Chen and coworkers identified that five urinary exosomal miRNAs (miR-194-5p, miR-146b-5p, miR-378a-3p, miR-23b-3p and miR-30a-5p) were significantly elevated in children with idiopathic nephrotic syndrome and markedly declined with improvement of the patients’ health status. Moreover, the concentrations of urinary exosomal miR-194-5p and miR-23b-3p were significantly correlated with urine protein content and other renal function indices. Thus, these two miRNAs could reflect the severity of pediatric nephrotic syndrome disease [69].

IgA nephropathy (IgAN) is a widespread primary glomerulonephritis that has been reported to be associated also with CD [70,71]. Szeto and colleagues examined the reliability of urinary miRNAs as biomarkers of IgAN [72]. In two different cohorts (i.e., discovery and validation cohorts) the authors identified 39 miRNAs significantly dysregulated in IgAN patients compared to controls. However, the authors focused only on four urinary miRNAs: miR-204, miR-431 and miR-555, which were significantly reduced, and miR-150, which was significantly increased in patients compared to controls. Interestingly, miR-204 alone achieved the best diagnostic accuracy among the pool of investigated miRNAs, reaching an AUC value of 0.976.

Acute kidney injury (AKI) is a complex syndrome that includes clinical manifestations ranging from minimal elevation in serum creatinine to anuric renal failure. Although there have been advances in research over the past few decades, the complex pathophysiology of AKI is not yet fully understood [73]. The regulatory mechanisms underlying post-AKI repair and fibrosis remain to be elucidated, although researchers have addressed the relevance of miRNAs only in the last few years. In fact, elevated levels of urinary miR-30c-5p and miR-192-5p in patients with AKI 2h after ischemia-reperfusion-induced kidney injury (IRI) underline the importance of these miRNAs as potential diagnostic markers for early IRI [74].

Finally, Ramezani and colleagues studied the correlation between circulating and urinary miRNAs in patients with minimal change disease (MCD) and focal segmental glomerulosclerosis (FSGS) [75]. The authors found that urinary levels of mir-1915 and miR-663 were downregulated in FSGS patients compared to MCD patients and controls, whereas miR-155 was upregulated. However, the authors concluded that the diagnostic and prognostic potential of these miRNAs warrants further study to use them as biomarkers in FSGS and MCD [71].

## 7. Diagnosis of CD and Adherence to GFD by Circulating miRNAs

Liquid biopsies, such as miRNAs circulating in the human serum/plasma (i.e., circulating miRNAs), have recently emerged as affordable and reliable biomarkers in many diseases [76,77,78], including CD [79]. This year, our group reported the discovery of novel circulating miRNAs as biomarkers for the diagnosis of CD and adherence to GFD by pediatric patients [23]. One of the aims of this study was to find reliable biomarkers able to avoid intestinal biopsies in children, especially those with serological levels of TGA-IgA <10 × the upper limit of normal, who generally undergo gastroduodenoscopy [17]. We found that a panel of circulating miRNAs can predict not only CD but that they are also able to return to the levels observed in control subjects after a strict GFD. We demonstrated the reliability and stability of these biomarkers (in particular, of miR-192-5p, miR-215-5p and miR-125b-5p either alone or in combination) and their potential in the clinical practice to help gastroenterologists in diagnosing and treating CD.

Our group also hypothesized a link between circulating and fecal miRNAs in intestinal diseases and the presence of an intricate network of interactions also involving the gut microbiota [80]. This is corroborated by a couple of seminal studies previously reported by others [81,82]. However, circulating and fecal miRNAs can represent only a face of the medal, as urinary miRNAs can complete this multifaceted picture (Figure 1).

We think that in the near future, urinary miRNAs could provide complimentary information not only to help clinicians to diagnose CD but possibly to assess adherence to GFD or the presence of comorbidities in CD patients.

## 8. Conclusions and Future Directions

We have discussed herein many papers dealing with the use of GIPs as biomarkers for monitoring adherence to GFD by CD patients. In the last decade, GIPs have been demonstrated to be reliable and stable biomarkers in many studies, although fecal GIPs could not significantly discriminate adult CD patients with persistent villous atrophy and those who recovered after a GFD for two years [83].

More recently, miRNAs have emerged as innovative diagnostic tools (i.e., liquid biopsies) that can be employed as potential biomarkers, as they have already been employed to diagnose many diseases, including CD. Larger cohorts are required to validate these results, as well as, in some cases, a confirmatory intestinal biopsy.

Considerable efforts have been made to find diagnostic methods to assess the presence of GIPs in feces and urine in order to evaluate the amount of ingested gluten or to monitor deviations from a GFD (i.e., adherence to GFD). the only non-invasive and direct methods reported in the literature in the last few years rely on the determination of GIPs either in feces or urine, although some of these determinations have been demonstrated to be dose- and time-dependent and only partially correlated to the interindividual kinetics of gluten processing and elimination [50].

Although by following strict methodological procedures (i.e., timing of sample collection, analytical sensitivity, extraction methods, etc.) we can assess the amount of GIPs in urine and feces and diagnose the accidental ingestion of gluten, the result of the test is a picture of a particular moment [83].

**Figure 2:**
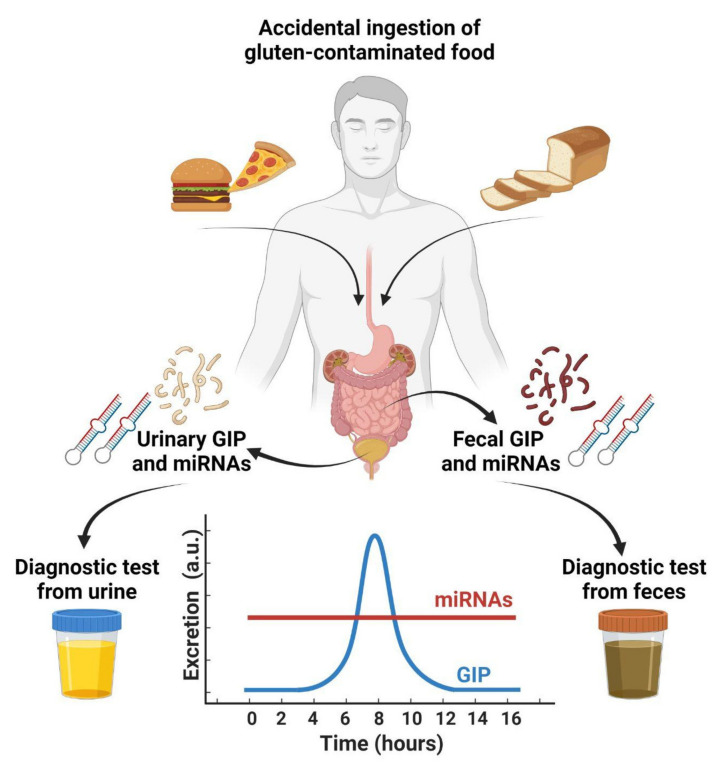
Accidental ingestion of gluten-contaminated food may lead not only to severe immunologic response but also to the production (by hydrolysis) of GIPs, which can be detected in feces and urine. The variability in GIPs concentration is time- and dose-dependent and follows a Gaussian curve. The evaluation of urinary miRNAs, their presence being linked to a particular tissue status, is stable almost until complete recovery or remission is achieved. This image was created with BioRender.com.

The time-course elimination of GIPs reported by some authors [84] follows a Gaussian curve (Figure 2).

The use of urinary miRNAs to obtain a picture of the pathologic condition of tissues (i.e., duodenum, glomerulus, kidney, etc.) is assumed constant for a quite long period until remission is complete, which, in the case of CD patients, occurs between 6 and 12 months and up to 2 years.

For all of these reasons, after having evaluated the reliability of circulating miRNAs, we support the study of urinary miRNAs not only to evaluate their ability as biomarkers of adherence to GFD in CD patients but also as a tool to monitor the status of the underlying tissues (i.e., duodenum, glomerulus and kidney), eventually corroborated by intestinal biopsies. Moreover, urine samples are more readily acceptable to patients compared to fecal samples due to the simplicity of collection and their transferability to the clinician.

Finally, further studies are needed to evaluate whether urinary miRNAs can predict the presence of intestinal/renal permeability and the concomitant presence of other comorbidities.

## Data Availability

Not applicable.
